# Haemoprocessor: A Portable Platform Using Rapid Acoustically Driven Plasma Separation Validated by Infrared Spectroscopy for Point-of-Care Diagnostics

**DOI:** 10.3390/bios12020119

**Published:** 2022-02-14

**Authors:** Kamal Prakash Prasanna Ravindran Nair, Thulya Chakkumpulakkal Puthan Veettil, Bayden R. Wood, Debjani Paul, Tuncay Alan

**Affiliations:** 1 Department of Mechanical & Aerospace Engineering, Monash University, Melbourne, VIC 3800, Australia; prasanna.nair@monash.edu; 2 Department of Biosciences & Bioengineering, Indian Institute of Technology Bombay, Mumbai 400076, India; debjani.paul@iitb.ac.in; 3 IIT Bombay—Monash Academy, Indian Institute of Technology Bombay, Mumbai 400076, India; 4 Monash Centre for Biospectroscopy, Department of Chemistry, Monash University, Melbourne, VIC 3800, Australia; thulya.chakkumpulakkalputhanveettil@monash.edu (T.C.P.V.); bayden.wood@monash.edu (B.R.W.)

**Keywords:** plasma separation, ATR-FTIR spectroscopy, point-of-care diagnostics, surface acoustic waves, open microfluidics, harmonic interdigitated transducers, particle sorting

## Abstract

The identification of biomarkers from blood plasma is at the heart of many diagnostic tests. These tests often need to be conducted frequently and quickly, but the logistics of sample collection and processing not only delays the test result, but also puts a strain on the healthcare system due to the sheer volume of tests that need to be performed. The advent of microfluidics has made the processing of samples quick and reliable, with little or no skill required on the user’s part. However, while several microfluidic devices have been demonstrated for plasma separation, none of them have validated the chemical integrity of the sample post-process. Here, we present Haemoprocessor: a portable, robust, open-fluidic system that utilizes Travelling Surface Acoustic Waves (TSAW) with the expression of overtones to separate plasma from 20× diluted human blood within a span of 2 min to achieve 98% RBC removal. The plasma and red blood cell separation quality/integrity was validated through Attenuated Total Reflection Fourier Transform Infrared (ATR-FTIR) spectroscopy and multivariate analyses to ascertain device performance and reproducibility when compared to centrifugation (the prevailing gold-standard for plasma separation). Principal Component Analysis (PCA) showed a remarkable separation of 92.21% between RBCs and plasma components obtained through both centrifugation and Haemoprocessor methods. Moreover, a close association between plasma isolates acquired by both approaches in PCA validated the potential of the proposed system as an eminent cell enrichment and plasma separation platform. Thus, compared to contemporary acoustic devices, this system combines the ease of operation, low sample requirement of an open system, the versatility of a SAW device using harmonics, and portability.

## 1. Introduction

Plasma constitutes about 55% of the whole blood, with the rest made up of Red Blood Cells, White Blood Cells, and Platelets. It is arguably the most important component in blood for disease diagnosis because it is the carrier of the circulating biomarkers that characterize many diseases, including cancer, Alzheimer’s, and infectious diseases [1]. Infrared spectroscopic techniques, coupled with exploratory tools, have played a pivotal role in human disease diagnosis including parasitic [2] and viral detection [3], and cancer diagnosis from various biofluidic samples [4]. Owing to the recent advances in biospectroscopy and availability of portable spectrometers, analysis of plasma can potentially be performed at the point-of-care (PoC), providing diagnostic results in under a few minutes after a plasma sample is deposited [2]. The traditional method for the separation of plasma from whole blood is centrifugation [5], wherein 2–4 mL of blood is spun down and the resulting supernatant is carefully collected by pipetting. While effective and highly reliable at the lab bench, this process requires a relatively large volume of blood per run and long processing times to facilitate practical extraction of plasma. Hence, centrifugation is not suitable for PoC applications when only small finger-prick volumes are available and/or the centrifuges lack portability.

Microfluidic devices have the potential to substantially reduce both the time taken to extract plasma from whole blood and the volume of blood required for the extraction process. Numerous microfluidic platforms have been proposed to perform either blood cell or plasma separation by both passive and active means [6]. Passive separation techniques commonly rely on microfabricated pillars, customized geometries [7,8] and porous filtration membranes [9]. These systems use the size-exclusion principle to separate the corpuscular components of blood to leave out the plasma in a continuous flow system.

Except for the use of one or more syringe/pressure pumps, passive separation techniques typically do not need an external actuator, but a major shortcoming of these techniques is that, while the microfluidic chip needs very small sample volumes (ca. a few µL), the pumps and the tubing used to drive the fluid flow in these closed devices increase the minimum sample volume that is required to a few ml. Furthermore, most ancillary equipment used with microfluidic chips are bulky, which limits their transport and use in the Point-of-Care scenario. Some of these problems have been addressed by paper-based platforms for the separation of blood plasma that can work with a drop of blood. One example of such a system is the μPAD, which was fabricated by patterning a chromatography paper with a solid ink (wax) printer and melting the ink to create hydrophobic barriers spanning through the entire thickness of the paper substrate. This was followed by surface functionalization for cell removal [10]. While these tests enable in situ colorimetric detection of analytes, they require additional biochemical reagents (e.g., agglutinating antibodies) and have a low separation efficiency. Surface Enhanced Raman Spectroscopy (SERS) measurements using paper substrates on diverse biological samples such as milk [11], serum [12] and teardrops [13] have been demonstrated. However, to date, very few examples have successfully managed to combine the spectroscopy of whole blood with paper-fluidic substrates [14]. Once the plasma is separated from blood on a paper-fluidic substrate, there is no provision for transferring it to another vial or microfluidic chip for further analysis.

However, active plasma separation from whole blood was demonstrated using magnetic, [15] dielectrophoretic (DEP) [16] and acoustic [17,18] forces to manipulate and segregate the formed elements. DEP uses electrodes to apply an electric field to induce differential migration of particles/cells based on their intrinsic charge bias within the said field. This does not require sample labelling but has only been used in the closed-system format to date. In contrast, magnetic separation techniques use functionalized magnetic beads in conjunction with a magnetic field for separation. Alternatively, acoustically actuated devices provide significant advantages for blood processing, since they use wavelengths that are close to the physical dimensions of cells [19]. Numerous groups used both standing [20,21,22] and traveling surface acoustic waves (TSAW) [23,24,25] for the effective manipulation of particles and cells in closed fluidic systems. SAW was used for open droplet-based sorting as well but given the manual loading of the devices, the consistent placement between loadings results in different process outcomes, thereby compromising repeatability.

**Figure 1:**
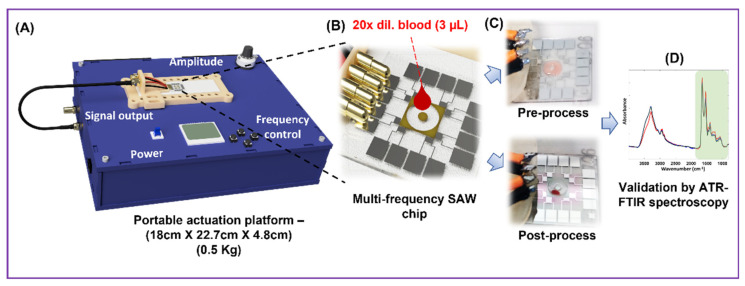
(**A**) Schematic diagram describing the process flow of the Haemoprocessor system for plasma separation. (**B**) The sample was first processed in the SU-8 process chamber. (**C**) The difference in the process chamber between pre-process and post-process. (**D**) Then, it was subject to IR spectroscopy for assessing the chemical composition of the obtained plasma.

To overcome these limitations, we developed Haemoprocessor; a portable, battery/line-power operated, label-free plasma separation system (Figure 1A), which uses travelling surface acoustic waves (TSAW) to process 3 µL of blood in under 2 min with 98% collection efficiency. It uses an open microfluidic system for ease of sample loading and collection. The Haemoprocessor consists of a piezoelectric LiNbO_3_ chip, on which eight interdigitated electrodes and a doughnut shaped process chamber are patterned (Figure 1B). The expression of overtones, along with multiple IDTs, allows this device to operate at multiple frequencies for the manipulation of particles of 2 µm–20 µm. After a blood sample was deposited in the chamber, a high-frequency oscillatory signal was applied to a selected electrode, which resulted in a TSAW. As the travelling wave interacted with the bound blood sample, the latter was spun around the chamber at a high velocity to collect all the blood cells in a ‘single lump’ inside the chamber (Figure 1C). The remaining plasma was then pipetted out of the chamber for subsequent analysis using ATR-FTIR spectroscopy (Figure 1D). We characterized the device performance in detail using both synthetic particle mixtures and human blood. ATR spectroscopic analysis of the plasma samples obtained from the Haemoprocessor were compared with those obtained from the ‘gold-standard’ technique, i.e., centrifugation, wherein excellent agreement was demonstrated, thus validating the quality of the plasma. Travelling SAW (TSAW), when used at a high power, can facilitate cell lysis, which can be used for DNA extraction for lab-on-a-chip applications [26]. Here, the actuation power is sufficiently low to prevent lysis and focus uniquely on the cell separation.

The entire “blood-in results-out” process is resolved in 2 min. The Haemoprocessor is easy to use, requiring little/no user skill aside from adding the blood sample and collecting the plasma. Finally, we present a custom-made self-contained actuation platform to generate the high-frequency signal, which weighs only ca. 0.5 Kg and occupies a volume of 18 × 22.7 × 4.8 cm^3^. The integration of Haemoprocessor with the actuation platform ensured that the complete separation system is easily transportable to any location for PoC applications, especially when there is a need to obtain high-quality plasma from small blood volumes such as with finger/toe pricks (as with infants, for example). This work also addresses the drawbacks with existing microfluidic systems, such as the lack of post-process characterization (to check for chemical alteration of the sample) and suitable statistical studies to assess repeatability, both of which brings it closer to a true PoC device than the ones reported to date.

## 2. Materials and Methods

### 2.1. Device Design and Fabrication

**Figure 2:**
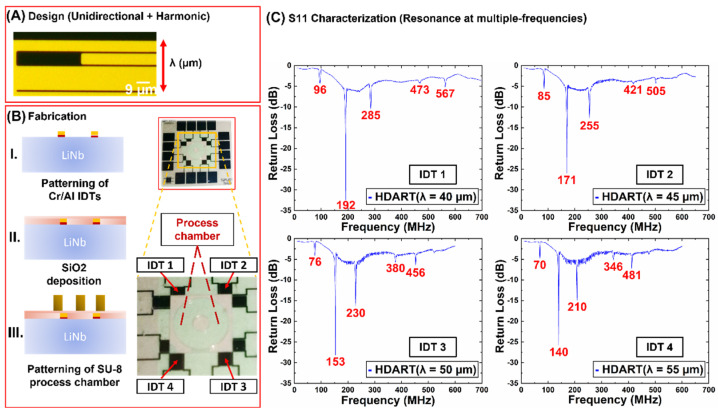
Figure depicting design, fabrication and the S11 characterization of the Distributive Array Unidirectional Transducers demonstrating harmonics (dubbed as HDART). (**A**) Top view of one set of interdigitated electrodes. Lambda (λ) indicates the wavelength. (**B**) Fabrication procedure of the Haemoprocessor system including the electrodes and the chamber. The HDARTs were designed and fabricated such that the wavelengths of 40, 45, 50, and 55 µm could be actuated on one device. (Note that another set of IDTs, at 90° to the first set, is also present, albeit not used in the following experiments.) (**C**) S11 characterization data of the HDARTs. Note the overtones expressed in each IDT type: the 1st and 2nd overtones were quite strong.

Figure 2A,B, show the fabrication of the Haemoprocessor devices on 128° YX LiNbO_3_ substrates with double-side polish (for optical transparency when used with an inverted microscope) using standard lithographic techniques. Photoresist (AZ1512HS) was spin-coated on 4″ LiNbO_3_ wafers, followed by patterning using a UV exposure tool (EVG6200). This was followed by metal deposition using e-beam evaporation (Intlvac Nanochrome II), wherein 10 nm/200 nm of Cr/Al was deposited on the patterned resist. The wafers were then placed in an acetone bath for resist ‘lift-off’, followed by IPA and DI-water wash. (Figure 2B). Then, a passivation layer (300 nm SiO_2_) was deposited using e-beam evaporation to mask the contact pads in case they become non-conductive. Lastly, a 45 µm layer of SU-8 3050 (Microchem) was spin-coated on the wafers, followed by UV exposure with mask alignment (EVG6200), post-exposure bake and development. The process temperatures were achieved by ramping (at 1.5 °C/min) to minimize the thermal stress on the LiNbO_3,_ as well as the SU-8. The completed wafers were then subject to dicing (DISCO DAD321) to obtain individual devices of the dimensions 16 mm × 16 mm.

**Figure 3:**
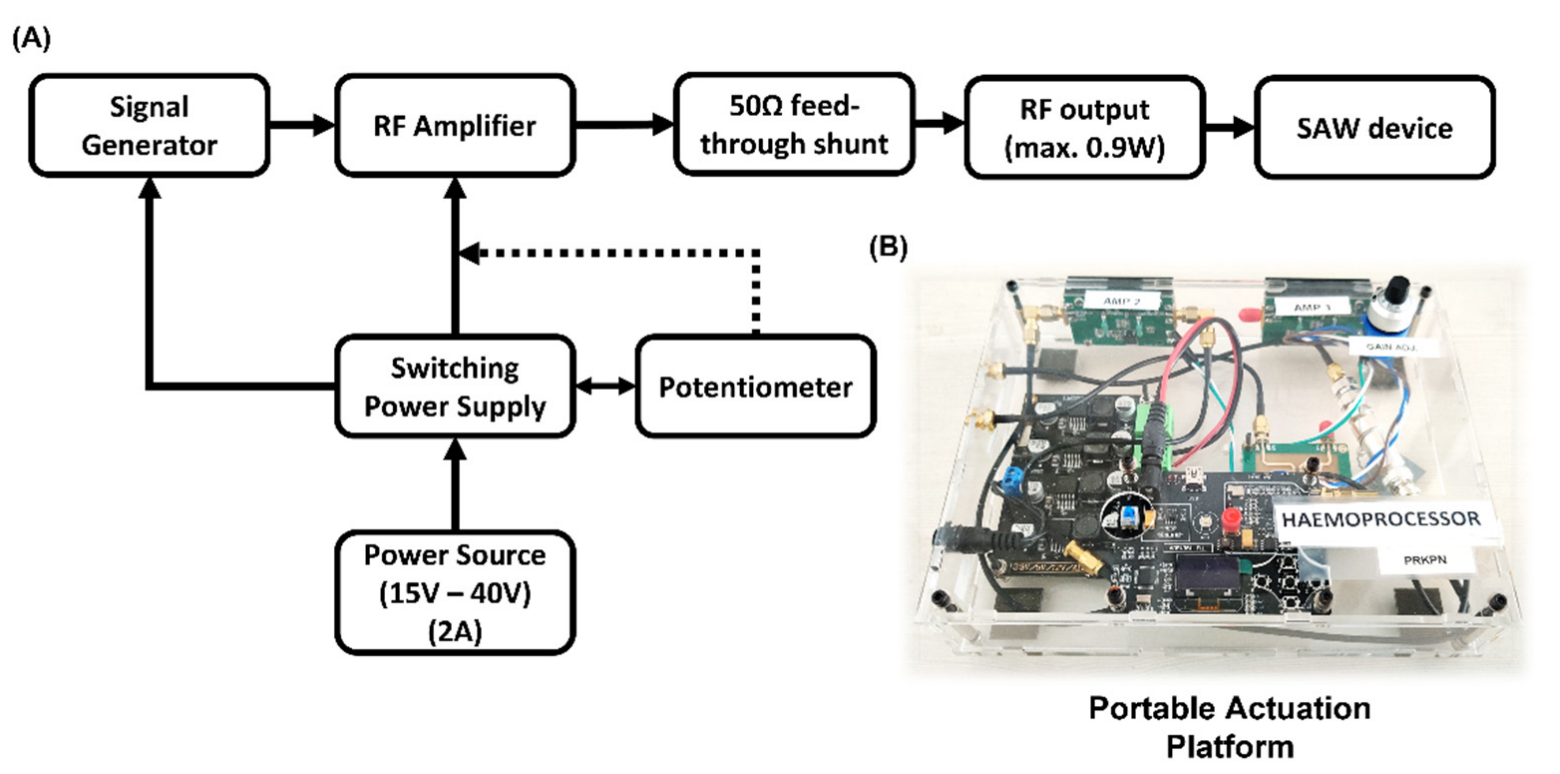
(**A**) The schematic for the portable actuation platform developed to be used with the Haemoprocessor chips. (**B**) The finished device weighs ca. 500 g and, as such, could easily be carried in a bag or a small briefcase. The use of the device was demonstrated in some of the particle and all the blood trials presented in this work. The performance of the platform is comparable to that of table-top generators with the additional advantage of portability.

The device layout and the frequency break-up of the devices are given in Figure 2C. Each chip (16 mm × 16 mm) has eight IDTs, of which only four are parallel to the crystallographic X-axis. Furthermore, each of the four IDTs comprised four different wavelengths (determined by the IDT period, Figure 3A)—IDT1 (40 µm), IDT2 (45 µm), IDT3 (50 µm), and IDT4 (55 µm). Note that the IDT metallization ratio was increased from 0.5 to 0.8 to manifest not only the fundamental frequency, but also the overtones. That said, the 1st and 2nd overtones were expressed quite strongly (Figure 2C), and these were used to actuate the device at different frequencies for the different technical replicates of a given sample type (henceforth, frequency hopping) runs described in Section 3.2 and 3.3. The central process chamber comprised a circular outer chamber with a square exterior, and a central pillar with open space in-between. The resulting fluid-space had a height of 45 µm, an inner radius of 1200 µm and an outer radius of 4000 µm.

### 2.2. Device Characterisation

The Haemoprocessor devices were subject to performance evaluation using the Return-Loss measurements to verify both the results of the design as well as the health of the device in question. Return Loss is a plot of the amount of energy transduced by the IDT in question versus the actuation frequency. The fundamental frequency was derived by the following equation

f= v/λ

where v is the velocity of SAW propagation on the Lithium Niobate crystal surface, *λ* is the wavelength (which is also the period of the IDT in question), and *f* is the frequency (while the SAW propagation velocity is 3988 m/s along the crystallographic X-axis on a pristine surface for the given substrate, the velocity in the device was less than that due to mass-loading effects).

Figure 2C depicts the frequency response of the said IDTs, wherein each ‘dip’ in the plot corresponded to frequency at which the IDT in question exhibited resonance (the greater the dip, greater was the efficiency). Note that each of the four IDTs demonstrated resonance at multiple frequencies, although the efficiency at each of these frequencies was not identical.

### 2.3. Development of a Customized Actuation Platform

As shown in Figure 3, the portable actuation platform comprised a RF Signal Generator ADF4351 (Analog Devices, Norwood, MA, USA), a RF power-amplifier, a power supply LM2596 (Texas Instruments, Dallas, TX, USA), a 10 kΩ potentiometer for RF power gain adjustment and finally, a 50 Ω feed-through shunt (RS components, London, UK) to protect the circuit from reflections due to impedance mismatch. The system could generate and amplify RF signals of frequencies ranging from 100 MHz to 700 MHz at powers ranging from 100 mW to 950 mW, the schematic of which can be found in Figure 3A. The whole system was installed and sealed in an acrylic casing, thus allowing for the easy transportation of the device (Figure 3B). The case was powered by a 15 V adapter plugged into 110–220 V line supply: alternatively, from 2 × 9 V alkaline batteries. This system was used to power the SAW devices in this project throughout, and it has proven to be stable and reliable to this end. The Haemoprocessor SAW chips were placed on a 3D-printed holder comprising a base, a mount with contact pins to interface with the SAW chip, and sub-miniature version A (SMA) wires and connectors to relay the RF input.

### 2.4. Experimental Protocol for Haemoprocessor

After mounting the Haemoprocessor chip and making the electrical connections, 3 µL of sample was loaded carefully into the chamber such that the sample did not merge over the pillar. The sample was actuated for 1–2 min, following which the plasma was pipetted out of the chamber for further analysis.

The post process clean-up was achieved by first rinsing the chip in DI water, followed by wiping with KIMWIPES(KIMTECH, Camberwell, VIC, Australia), and finally rinsed with isopropyl alcohol. The chips were air-dried before their next use, using an N_2_ air gun.

### 2.5. Particle Collection Trials with Polystyrene (PS) Beads

Polystyrene beads of sizes 6 µm (Duke Scientific Corp, Freemont, CA, USA) and 9.9 µm (Duke Scientific Corp^®^), both green—fluorescent; these were diluted in 2% PEG solution to a concentration of ∼150 particles/µL. For the PS bead mixture runs, beads of sizes 2.1 µm (Magsphere Inc., Pasadena, CA, USA), yellow—fluorescent; and 5 µm (Duke Scientific Corp), green—fluorescent; were also diluted and intermixed in 2% PEG solution to a concentration of ~200 particles/µL of 5 µm and 1000 particles/µL of 2.1 µm. The load volume was 3 µL and the devices were actuated using the portable system at a constant power of 0.5 W. The process was visualized using an upright microscope (BX43, Olympus, Tokyo, Japan) and simultaneously recorded (B872—Pixelnc., Ottawa, ON, Canada) with the appropriate fluorescent filters. The images from the collection runs were processed using an image-processing software (ImageJ-NIH, Bethesda, MD, USA), the figures were tabulated using Microsoft Excel (Microsoft, Redmond, WA, USA), and the graphs were plotted in Origin Graphing Software 2018 (OriginLab Corp, Northampton, MA, USA). A total of three sets of collection data were obtained for each frequency of actuation (at constant power of 0.5 W) and then averaged to plot the collection (%) graphs.

### 2.6. Processing of Whole Human Blood

Whole blood was obtained from the Australian Red Cross Blood Bank (Melbourne, VIC, Australia) and screened for standard pathogens prior to analysis. Prior to experiments, blood samples were stored at 4 °C and used within two weeks. The blood samples were diluted by 20× with phosphate buffer saline (PBS) solution (pH 7.4) for plasma separation using Haemoprocessor and further processed for spectroscopic analysis as explained in 2.6. Blood samples for centrifugation were diluted 20× with 0.9% NaCl solution (Baxter, Deerfield, IL, USA) for spectroscopic analysis. The SAW chips were driven using the same setup described in Section 2.5, except that the power set to 0.63 W.

### 2.7. ATR-FTIR Spectroscopy on Whole Human Blood

The spectroscopic measurements were performed using a single bounce ATR–FTIR spectrometer (Alpha FTIR Spectrometer, Bruker Corporation, Billerica, MA, USA). The spectral measurements were recorded in the 4000 cm^−1^−800 cm^−1^ range at a resolution of 8 cm^−1^ with a zero-filling factor of 2. The background (measured before every replicate) and sample spectra were recorded with 64 co-added interferograms. Plasma samples (1–1.5 µL) separated from the Haemoprocessor device, were actuated at 285 MHz (the best performing frequency from the PS bead collection runs) were placed onto the ATR crystal and dried. For spectroscopic analysis using the Haemoprocessor device, four biological replicates were used to assess the repeatability and reproducibility of the proposed approach. For centrifugation experiments, whole blood was diluted 20× with saline solution. The plasma fraction was isolated at 2000 g for 5 min using centrifugation (HERAEUS, Pico 17 microcentrifuge, ThermoFisher Scientific, Waltham, MA, USA). Approximately 2 µL of centrifuged plasma was spectroscopically analyzed using the same spectral parameters. The spectroscopic data were processed and evaluated using MATLAB 9.4 Release2018a (Mathworks, Natik, MA, USA) software package and PLS-Tool Box (Eigenvector, Manson, WA, USA). The raw spectral data were processed by baseline correction and weighted normalization using an automatic weight least-squares algorithm of an order of 2 and standard normal variate (SNV), respectively. Second derivatives were calculated using the Savitzky–Golay algorithm convolved to a 11-point smoothing function with a 3rd-order polynomial.

### 2.8. Principal Component Analysis (PCA)

PCA (MATLAB 9.4 Release2018a (Mathworks, Natik, MA, USA)) software packages and PLS-Tool Box from Eigenvector (Manson, WA, USA) was applied to the whole spectral dataset, obtained from both centrifugation and SAW device and pre-processed as stated above. Five principal components (PCs) were used to decompose the data.

## 3. Results and Discussion

### 3.1. Process Mechanism and Flow Dynamics

The Haemoprocessor uses a travelling SAW (TSAW) [27,28,29] generated by applying a high frequency AC voltage to one of the eight IDTs patterned on the LiNbO_3_ substrate at their designated frequencies. When this ‘out-of-plane’ surface wave traveling on the substrate reached the fluid volume bounded by the doughnut shaped open track, it began transferring mechanical energy to the liquid media and generated a rapidly decaying pressure wave propagating within the fluid at the Rayleigh angle in the out of plane direction [30,31,32]. The attenuation of the pressure field in the bulk of the fluid, as well as along the fluid-substrate interface, resulted in a spatial variation in the body forces acting in the fluid and generated an acoustic streaming field [33,34].

**Figure 4:**
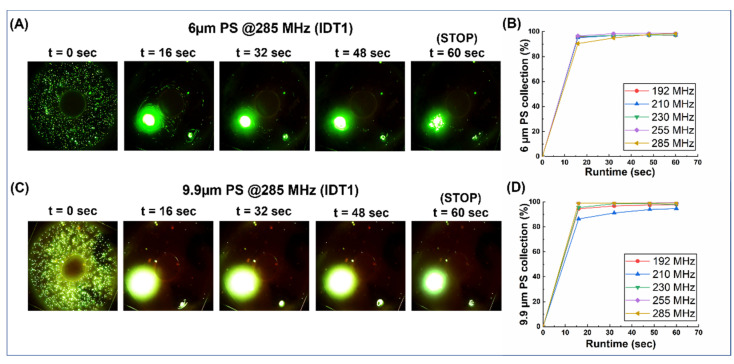
The frequency hop results from the collection of 6µm and 9.9 µm polystyrene beads with a load volume of 3 µL. It was noted that these particles showed a greater degree of settling at the bottom of the process chamber when compared to other sizes but, nevertheless, the collection showed a marked improvement at higher frequencies. (**A**,**C**) Snaps from the collection videos of 6 µm and 9.9 µm (respectively). (**B**,**D**) graphs plotting the collection (%) vs. time for 6 µm and 9.9 µm (respectively).

**Figure 5:**
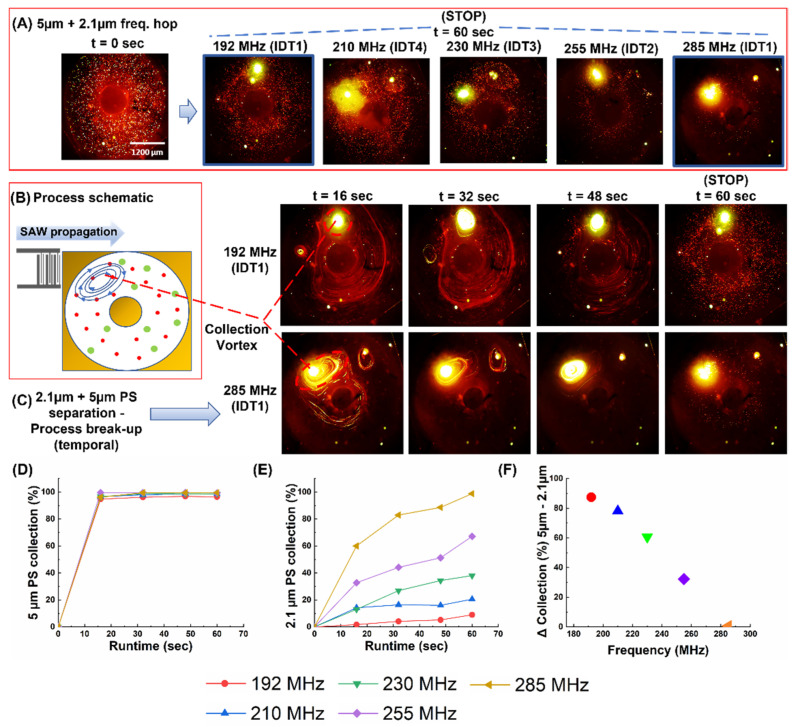
Depicting the sorting of 2.1 µm particles from 5 µm polystyrene beads. (**A**) The result of the particle solution after 60 sec of actuation at 192 MHz, 210 MHz, 230 MHz, 255 MHz, and 285 MHz (**B**) Pictorial representation of the result of the interaction between the TSAW and the particle solution. (**C**) The process break-up with 16-sec intervals at 192 MHz (above) and 285 MHz (below) to contrast the difference in collection from end-to-end of the frequency range. (**D**) Plot of collection (%) of 5 µm PS beads versus time. (**E**) Plot of collection (%) of 2.1 µm vs. time across all frequencies (**F**) Plot depicting the difference in collection efficiency (5 µm−2.1 µm) vs. frequency. (Refer to SV1 for real-time videos of one set of the trials at 192 and 285 MHz).

The pressure field pushed the liquid at the center of the wavefront, thereby creating a void that was quickly filled by the surrounding liquid. With constant bombardment by the acoustic waves, the void-filling action by the liquid typically results in a dual vortex on both sides of the wavefront. The spatial gradients at the edges of the incoming beam generated an acoustic force, which tends to translate the particles further away from the center of the incoming beam, towards the inner streamlines of the vortices, with each attempting to attract the particles into itself. Since the acoustic force acting on each particle is proportional to its volume [35], the large particles were pushed towards the center of the vortex, where they could be concentrated [36]. In the present case, the IDTs were placed in such a manner that only half of the wavefront was exposed to the process chamber. This resulted in the formation of only a single vortex at the edge of the fluid, which, in turn reduced the number of aggregation points of the particles to one (as seen in Figure 4A,B and Figure 5A,C). While the largest particles were trapped in the spirals of the vortex, particles with diameter smaller than a threshold value escaped the vortices and continued following the external streamlines, subject to the translational drag force from the flow of the liquid (throughout the path) [37]. In the present case, as discussed in the results section, this resulted in the smallest particles flowing along the circular track. The collection of smaller particles could be accomplished by dialing the actuating frequency up, which results in larger body forces (*F_b_∼ω*^2^) and further increases the body force gradient and, as a result, the acoustic forces [35,38].

The use of TSAW for the separation of cells has been extensively reported using closed systems, which typically require sample volumes larger than 100 µL, and active pumping elements and tubing components should be employed to move the liquids within the microfluidic channels. [17,28,29,39,40,41,42,43,44]. These additional complexities, which are easy to accommodate at the lab bench, limit the uptake of the devices in point-of-care settings (none of the earlier devices reported a portable battery-operated platform). In contrast, when open systems consisting of sessile droplets deposited on a blank piezoelectric substrate were used, the fluid interface and the complex flow profiles within the droplet limit the separation effectiveness in exchange for operational simplicity.

Haemoprocsessor addresses all of these issues using a doughnut-shaped process chamber [45], which was designed to not only make the exposure of the SAW on the liquid more consistent, but also to also ensure that the flow was constrained, both of which ensured that the collection zone, along with the rate, was consistent between runs. The height of the device was restricted to 45 µm to reduce the influence of the vortices in the out-of-plane direction and this, in turn, simplified the collection process in that virtually all the collection took place in a single location. The combination of these factors allowed the device to show good repeatability with respect to collection efficiency and process time. The plasma derived from Haemoprocessor was subject to multiple replicates (*n* = 9) of post-process analysis using ATR-FTIR spectroscopy, to validate that the quality of the obtained plasma was comparable to that obtained from centrifugation. The devices do not need any external pumping, tubing and are powered by a “shoebox” sized battery-operated driver weighing only 500 g, hence making the Haemoprocessor clearly stand out from the rest of its counterparts.

In this course of work, the acoustic wavelengths used were 20 µm (192 MHz), 18.3 µm (210 MHz), 16.6 µm (230 MHz), 15 µm (255 MHz), 13.3 µm (285 MHz). As was demonstrated in the trials reported in the following sections, the shorter wavelengths showed better collection, especially in case of the smaller particles. That said, the decrease in acoustic penetration depth with increasing frequency resulted in unstable vortices, leading to the inability to retain captured particles. Therefore, for a system with a given dimension, the ‘sweet-spot’ frequency was that offering maximum collection without falling short of creating a vortex due to insufficient fluid penetration. On the other hand, the scaling-down of the process chamber (although not practical for an open system with manual loading, such as the Haemoprocessor) would have enabled the entrapment of particles of even smaller sizes [38]. The interaction of particles of different sizes when subject to TSAW fields of different frequencies are discussed in the following sections.

### 3.2. Frequency Hopping and Its Effect on Collection Efficiency for 6 µm and 9.9 µm PS Beads

The Haemoprocessor devices were first used to assess the behavior of polystyrene beads of different sizes loaded in the device’s process chamber and the device was actuated at 12 different frequencies (70,76,85,96,140,153,171,192,210,230,255, and 285 MHz) as part of 12 trials, each with at least three technical replicates. For each case, a continuous video of the process was recorded for 60 s, after which the actuation was stopped (Figures S1 and S2). Snapshots of the video at a 16-s interval were then processed to estimate the collection efficiency at each time interval for both 6 µm (Figure 4A) and 9.9 µm (Figure 4B) PS beads.

Optical micrographs were obtained at intervals of 16 s, and this was carried out for the whole process (lasting 60 s). The snaps were processed for particle counts, the collection percentage was calculated for each time interval, and then plotted for both 6 µm (Figure 4A,B) and 9.9 µm (Figure 4C,D) as runtime vs. collection (%). For frequencies below 171 MHz, both 6 µm and 9.9 µm particles show little or no aggregation (data not shown) or the collection behavior was not consistent (data not shown). From 192 MHz and beyond, >90% of both particles were collected within the first 16 s of the run, and the location of the collection was consistent between runs and, therefore, was used to quantify the collection (%). However, when the entire run was compared between the two particle sizes across the five frequencies where aggregation was noted, the collection efficiency improved (albeit in small increments) as the frequency was increased (Figure S3), which was particularly true of the particles that tend to stick to the bottom of the process chamber, since they demonstrated the least velocity due to the no-slip condition. As for the effect of process duration, the collection (%) flattened out and, at process stop (t = 60 s), the graph was practically flat. This was true for all actuation frequencies and, thus, it became redundant to continue the run past this point.

### 3.3. Frequency Hopping and Its Effect on Collection Efficiency Mixture of 5 µm + 2.1 µm PS Beads

As a follow-up to the previous study, a solution containing 2.1 µm and 5 µm PS bead mixtures were subjected to the same set of frequencies as the trials with the 6 µm and 9.9 µm PS beads. (Note that, in all these trials, factors such as load volume, amplitude, and runtime were kept constant). As before, the process was stopped at intervals of 16 s until cessation at 60 s. The snaps from each of the time intervals (in triplicate) were used to calculate the collection efficiency of both particles (Figure 5A–C). The collection efficiency for both increased with the actuation frequency, but the increase for the 2.1 µm was over a wide range: ca. 10% at 192 MHz vs. ca. 98% at 285 MHz. The collection of both the 2.1 µm and 5 µm polystyrene particles was found to be directly proportional to the frequency of actuation (Figure 5D).

An interesting point was that the collection (%) of the 9.9 µm, 6 µm, and 5 µm particles were similar in that within the first 16 s: more than 85% of the particles were collected. However, the collection of the 2.1 µm particles stood apart from the rest, seeming to be much more sensitive to the actuation frequency (Figure 5E), even though the other three also showed an improvement (albeit marginal) in collection (%) with an increase in the actuation frequency. As for the effect of the process duration, once again, the collection trend for the 5 µm was similar to that of the 6 µm and 9.9 µm. However, for the 2.1 µm particles, the graph was steadily climbing throughout the process, which indicated that the collection rate was more consistent in this case. For instance, after 16 s of actuation, the collection efficiency of 2.1 µm particles at 192 MHz was practically zero, while, at 285 MHz, the efficiency increased to ca. 60% (SV1). As seen in Figure 5F, increasing the actuation time to only 60 s results in a significant difference in the collection efficiencies between both particle populations. This differential collection was highest at 192 MHz, and gradually diminished as the actuation frequency was increased to 285 MHz. For the latter, the flows within the channel were strong enough to concentrate both particle populations, which could be exploited to rapidly remove particles of both sizes. Furthermore, this trend was carried forward across all time intervals with the increase in the actuation frequency, which resulted in a corresponding increase in collection (%). However, note that it was not a gradual trend that grew in prominence with a decrease in particle size, but a phenomenon that manifested dramatically at the smallest size under consideration and this, in turn, pointed to a threshold size at which the said change in collection behavior becomes apparent.

### 3.4. Filtration of RBCs from 20× Diluted Blood at 285 MHz/0.63 W

**Figure 6:**
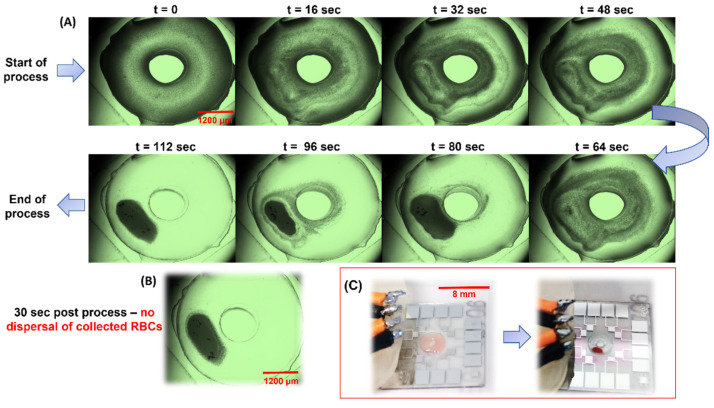
(**A**) The results from running 1:20 human blood at 285 MHz with a load volume of 3 µL. Starting from the top-left (t = 0 s), each window represents a time difference of 16 s from the previous one. (**B**) Image snaps of the device pre-run and post run. Note the mass of RBC does not diffuse back even 10 s after device stop. (Refer to SV2 for real-time videos of one set of the trial). (**C**) The working of the device when seen with the unaided eye—note the clear accumulation of the RBCs leaving behind the clear fraction that could be extracted using a micropipette.

**Figure 7:**
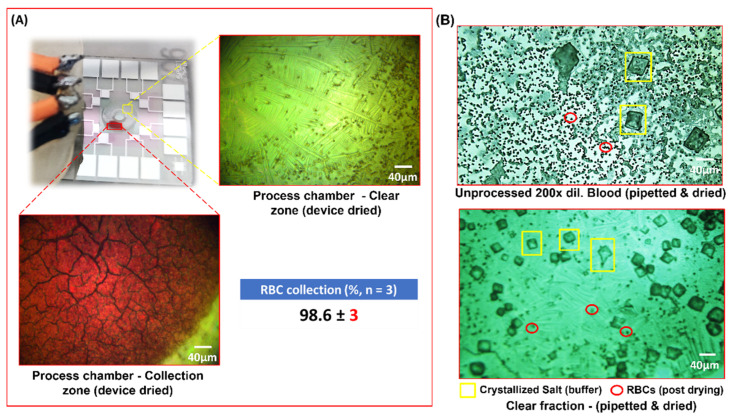
(**A**) Depicts the device post process of 20× diluted human blood. The deep-red patch was the area of accumulation of RBCs whereas the remaining zone appeared transparent. Note the 20× magnified images of the said zones post drying of the sample in the device. (**B**) Depicts the post-drying images from pipetted drops of the unprocessed samples (top-right) and processed 20× human blood (bottom-right). Note that the top-right image was imaged at 200× dilution since the cell density in a 20× diluted but unprocessed blood was too high and, therefore, would lack any discernible features.

Human blood diluted 20-fold in Phosphate Buffer Saline (pH 7.4) was subject to the sorting process mentioned in the previous sections. At this dilution, the average cell count was 250,000 cells/µL and, with a 3 µL load volume, the total number of RBCs in the Haemoprocessor device was ca. 0.75 million cells. The typical size of RBCs in an otherwise healthy individual is 6–8 µm. Contrary to the runtimes for the PS bead collection experiments, the runtimes here had to be extended to 2 min owing to the non-Newtonian behavior of blood, as well as the discoid shape of RBCs, resulting in significant differences in the physical properties and, in turn, the cell migration in the acoustic field. The frequency was set to 285 MHz since this was the best-performing of the PS bead collection runs. Aside from the increased runtime, the course of the process was as expected for a particle with a cross-section of 6–8 µm (Figure 6A). The power was increased to 0.63 W due to the difference in the viscosity of the blood sample, although it was diluted from the 2% PEG solution used to suspend the PS beads. Like the PS bead trials, the process was recorded, and snapshots were taken at regular intervals, starting from *t* = 0 s, at intervals of 16 s till device stop at 120 s (not shown). The complete run can be accessed at SV2. Given that these runs involved live cells and it was imperative to retain their viability throughout the process, the temperature of the device was continually monitored. At 285 MHz and 0.63 W, the temperature of the process did not exceed 36.3 °C even after 2 min from *t* = 0 of the collection trials (the temperature readouts for different frequencies and amplitudes were plotted in Figure S4). The RBCs were visualized in brightfield mode and, since they were naturally pigmented, no staining/labelling was needed to track them during the process. Furthermore, the collected mass of RBCs did not diffuse after stopping the process (Figure 6B), thus allowing for the practical use of fractions resulting from the device for further analyses. The RBC collection was evident even without the use of a microscope (Figure 6C and Figure 7A). This added to the simplicity of the setup, in that the user could retrieve the clear liquid using a micropipette (as was performed for the spectroscopic analysis of the same).

The collection efficiency of the runs was calculated by allowing the processed sample to dry in the chip. Snaps from five random locations of the clear region were used to count the number of cells, and this was performed in three technical replicates. The efficiency came to ca. 98% with a Standard Deviation (henceforth, SD) of three (Figure 7A). The HCF was also pipetted to a blank LiNbO_3_ slab and compared to similarly pipetted 200× diluted unprocessed blood (Figure 7B). Note that the counts used to calculate the collection efficiency was from the device’s post-processing (which includes the cells that settled at the bottom), and yet the device yielded a high collection (%). Note the large cuboidal crystals in the frame, which were the result of crystallization of the salt from the Phosphate Buffer Saline. The comparison of the clear fluid or Haemoprocessor-derived Clear Fraction (henceforth, HCF) from this process, with plasma isolated from centrifuged blood, was carried out spectroscopically and is discussed in the subsequent sections. The Haemoprocessor-derived RBC Fraction (henceforth, HRF), on the other hand, is compared with the RBC fraction from centrifugation using spectroscopic analysis in the following section.

Assessments of the platform’s effectiveness at other dilution trials were performed using 10× diluted Human Blood, but the high density of blood cells substantially reduced the effectiveness of the device (Figure S5). In the extreme case of undiluted blood, the acoustic waves had a very limited discernable effect due to the very high number of cells.

### 3.5. Spectroscopic Comparison between Centrifuged Plasma and Device-Derived HCF

**Figure 8:**
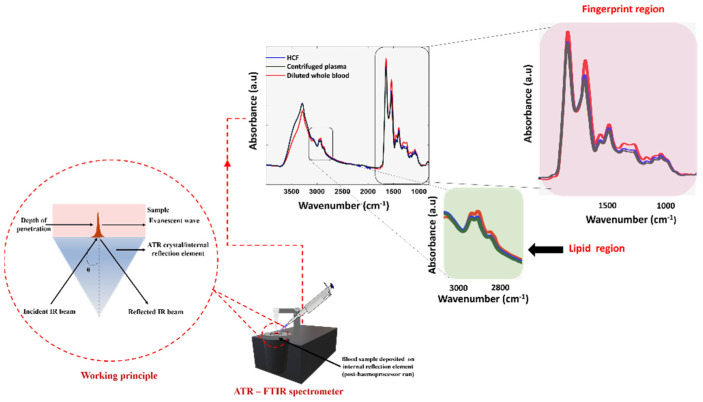
The ATR-FTIR measurements showing the raw spectrum of HCF, centrifuged plasma, and diluted whole-blood.

The plasma fraction obtained after centrifugation of the 20× diluted human whole-blood and the HCF were spectroscopically analyzed using the ATR-FTIR technique. The centrifugation method is regarded as the ‘gold-standard’ technique to separate the molecules from a solution relative to their size, shape, density, and viscosity of the medium, and rotor speed was used to compare the purity of the HCF. Post actuation of the Haemoprocessor, the diluted whole blood was segregated into HRF and the HCF containing serum. The spectrum of both the red blood cells and sera were immediately measured after their separation. Figure 8 shows the pre-processed spectra of both the dried centrifuged plasma and the dried HCF, demonstrating the similar biochemical signatures, as expected, in regions including 3100−2800 cm^−1^ (lipid region) and 1800−1000 cm^−1^ (fingerprint region).

The drying process was introduced to eliminate the strong contribution of water, often observed at around the 3300−3100 cm^−1^ and 1700−1600 cm^−1^ region. Since the phosphate moieties from PBS solution presumably interfered with the spectrum, it was ideal to use saline as the solvent.

### 3.6. Variance Study

**Figure 9:**
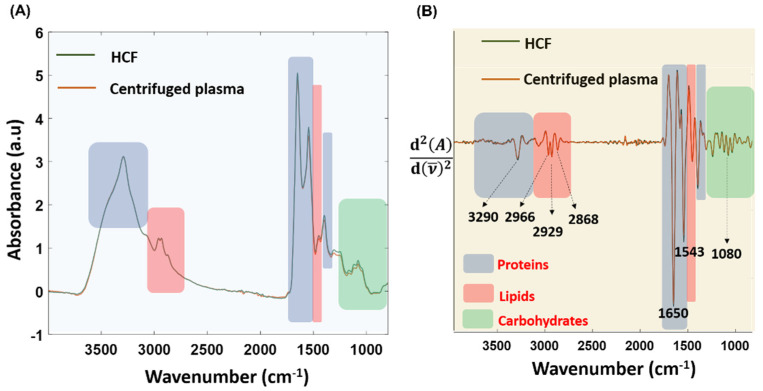
(**A**) The baseline-corrected and -normalized averaged spectra from HCF and centrifugation approaches. (**B**) Corresponding second-derivative spectra.

The regions corresponding to carbohydrates, proteins, lipids, and fatty acids are highlighted in the raw (Figure 9A) and second derivative spectra (Figure 9B) derived from both methods. This indicated that Haemoprocessor was able to successfully isolate the clear component from the HRF. The stronger C-H stretching bands in the 3100−2800 cm^−1^ region from the HCF were indicative of lipid moieties, which appeared in higher abundance in serum compared to red blood cells. Additionally, the spectra obtained from the HCF were very similar to the spectra acquired from the ‘gold-standard’ centrifugation method in both regions (3100−2800 cm^−1^ and 1800−1000 cm^−1^).

The technical replicates from four biological replicates of HCF and two biological replicates of centrifuged plasma were averaged after baseline correction, and the variance was evaluated using the standard deviation (SD) function for the whole spectral region of 3900−800 cm^−1^. The average SD between pre-processed spectrum from the HCF and centrifuged plasma was found to be 0.156 and 0.097, respectively. The largest variance between the HCF spectrum was observed at 1535 cm^−1^, with an SD of 0.568, and the lowest variance appeared at 3814 cm^−1^, with an SD of 0.0067. Similarly, the largest variance between centrifuged plasma spectrum was observed at 1658 cm^−1^, with an SD of 0.43 and lowest variance was at 3797 cm^−1^, with an SD of 0.009. The SD values from the centrifuged plasma spectrum were minimal compared to the HCF spectrum. However, the measurements from the technical replicates of four biologically distinct samples obtained from Australian Red Cross blood bags were used to build the SD model of HCFs, whilst data from all technical replicates of the two biological replicates were employed for the centrifuged-derived plasma. Hence, the slightly higher SD of HCFs could be attributed to the higher number of technical and biological replicates as compared to centrifuged plasma. In other words, despite the higher number of technical and biological replicates (which can influence diverse conditions such as temperature and humidity), variance between HCFs was comparable to centrifuged plasma.

### 3.7. Assessing and Discriminating the Spectral Data Using PCA: A Chemometric Investigation

**Figure 10:**
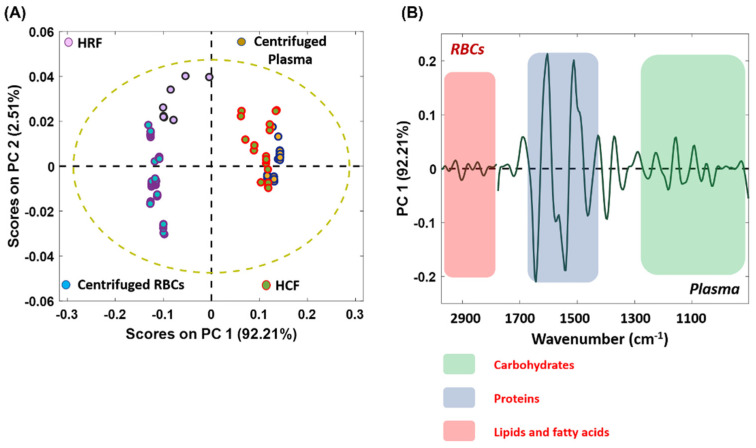
(**A**) 2D PCA score plot on the dataset consisting of HCF, HRF, plasma and RBCs from centrifugation method and, (**B**) the corresponding loading plot.

PCA is a powerful, unsupervised, multivariate chemometric approach based on finding linear correlations between the original variables using PCs and the mode of orthogonal transformation, which decomposes the dataset into linear combinations of the original variables. It relies on a dimensionality reduction strategy and is applied to large datasets to identify and preserve the variability among the variables [46]. Herein, the method was adapted to find linear correlations between the spectral data secured from both centrifugation and Haemoprocessor runs. The spectral variance across the subpopulation of RBCs and plasma from different methods over a 3000−1000 cm^−1^ region was processed using this model. Figure 10A shows the two-dimensional (2D) scores plot (PC1 vs. PC2), while. Figure 10B shows the corresponding PC1 loadings plot. From the significant positive PC1 values, it was evident that the data from the centrifuged plasma were well clustered with the data of HCFs. This implied that there was biochemical homogeneity between the two methods. This variance could be further visualized using a loadings plot, which denotes the variables or components that are most responsible for the clustering pattern observed in the scores plot. Since PCA is performed on the second derivative spectral data, positive loadings correspond to the negative scores and vice versa [47]. Herein, the negative loading is associated with the major contributions from centrifuged plasma and the HCF, whilst the positive loading denotes the influence of RBCs. The CH stretching vibration region (3000−2800 cm^−1^, acyl chain lipids) appeared to be more pronounced in the negative loadings compared to the positive loadings, which justifies the increased level of proteins and lipid contents in human plasma and HCF. Similarly, the COO^–^ stretching vibration from amino acids was observed in the 1410−1400 cm^−1^ region of plasma. The 1170−1000 cm^−1^ region is attributed to contributions from carbohydrates and phospholipids. The contribution of circulating free deoxyribonucleic acid (DNA), present in plasma and lymphocyte-associated DNA (very minor component), was minimal in the samples. It has been reported that the DNA content in healthy controls varied widely, and is in the nanogram range per ml of serum samples [47,48]. Moreover, because the blood was diluted 20× with saline solution, the spectroscopically detectable range of nucleic acids was presumably further reduced. Hence, the region 1250−1000 cm^−1^ could be assigned to the contributions from carbohydrates and phospholipids. The centrifuged RBCs and HRF were less correlated and formed more distinct clusters compared to the serum HCF.

This could be the result of residual contamination from the sera attached to red blood cells in the HCFs. The PCA results confirm the biochemical homogeneity between HCF and centrifuged plasma samples. The fact that the two different datasets are indistinguishable on the PCA scores plot indicates that the datasets are highly correlated. This is also confirmed in the average second derivative spectra of the serum samples (Figure 9A). Since identical pre-processing conditions, such as sample preparation and spectral acquisition parameters, were maintained throughout the experiment, the remarkable similarity of the plasma and clear fraction groups demonstrates the effectiveness of the Haemoprocessor to separate purified plasma from a micro-sample of whole-blood using finger prick volumes of whole-blood.

### 3.8. Potential Applications of the Platform

The Haemoprocessor platform enables the user to perform an operation that is fundamental to blood-based diagnostic tests: plasma separation. The platform can process 3 µL of sample in 2 min while being portable and easy to use (open system) with minimal ancillary equipment. Although blood needs to be diluted 20-fold before use, the platform will prove useful in cases where only small draw volumes (<50 µL), typically collected by finger (or heel) prick methods, have to be processed. Capillary blood sampling by prick method is a simpler and faster procedure when compared to venous blood sampling. The procedure is recommended when running tests on infants and newborns, especially when an illness has resulted in them being subjected to multiple blood tests. Amongst adults, it is also useful in patients with severe burns, extremely obese patients, geriatric patients, individuals who are anxious about sampling, patients with a tendency toward thrombosis, patients whose surface veins need to be preserved for intravenous therapy, and patients with fragile or inaccessible veins. Yet another advantage would be in the case of PoC testing, wherein finger-prick blood is preferred over blood drawn from an artery since the latter demands more expertise on the user’s part. In a typical finger prick, the amount of blood drawn is ca. 10 µL, which is less than what is required for any centrifuge based separation technique [49]. On the other hand, the Haemoprocessor platform is suitable for use in both these cases since, post-dilution, the total sample available for processing would be ca. 200 µL.

### 3.9. Importance of ATR–FTIR Spectroscopy as a Potential Tool in PoC Settings

ATR–FTIR spectroscopy has been widely applied in biomedicine to interrogate various specimens, including blood and blood-derived products [50,51]. For example, a large field trial conducted in Thailand resulted in a sensitivity comparable to the polymerase chain reaction (PCR) assay utilizing a cloud-based data management system for parasitemia detection and quantification. The technology is highly sensitive with low logistical requirements due to its portability, making it a game-changer in the context of malaria elimination programs [2]. Reducing the sample volume from 3 mL blood to fingerpick volumes facilitates the translation of the technology to PoC settings, making it especially applicable to infants, who are the major victims of malaria. Thus, combining a state-of the art Haemoprocessor and commercially available portable ATR-FTIR spectrometer provides major advantages compared to other competing technologies in resource-limited settings.

## 4. Conclusions

In this work, we have presented a portable, open-microfluidic, plasma-separation platform, which comprised a multi-frequency TSAW chip with an actuation device that allowed for the generation of RF signals from 100 MHz to 700 MHz with adjustable power (100 mW–900 mW). This was able to separate plasma from 3 µL of diluted 20× whole blood within a time span of 2 min. The resulting plasma was subject to microscopy, and the RBC count of the same confirmed the removal of ca. 98% of cells. Further analysis of the fraction using IR spectroscopy indicated that the chemical composition was practically identical to the plasma obtained from centrifugation. Lastly, the difference in the standard deviation of fractions from multiple runs between the centrifugation and Haemoprocessor device was minimal, indicating that the repeatability of this platform is comparable to the ‘gold-standard’ technique for plasma separation, that is, centrifugation.

With regards to the ergonomics of the platform, the case and the ancillary equipment are ca. 0.5 kg and, therefore, easy to carry. The relatively small dimensions also make it the size of a medium-sized book; thus, it could be carried by hand or in a college bag/briefcase. The visibility of the process by the unaided eye obviates the need for ancillary tools to perform plasma separation (such as syringe/pressure pumps) to aspirate the sample, as a standard micropipette will suffice. The Haemoprocessor could be used in conjunction with similar small-footprint devices such as miniature spectroscopes/microscopes to conduct tests post-process. The ability of this platform to generate plasma for any downstream process involving small sample volumes, such as PCR, IR spectroscopy, Gel electrophoresis et cetera, will be an asset, especially when running tests at remote locations, or when the availability of the sample is limited (as in blood draws from infants). Having demonstrated the ability to collect cells and given its range of operation with regards to the possible frequencies of actuation, the device has potential use in other open-fluidic cell sorting operations.

## Data Availability

Not applicable.

## Supplementary Materials

The following are available online at https://www.mdpi.com/article/10.3390/bios12020119/s1. Figure S1: Frequency hopping summary for 6 μm PS beads; Figure S2: Frequency hopping summary for 9.9 μm PS beads; Figure S3: Frequency hopping collection (%) plots for 6 μm and 9.9 μm PS beads; Figure S4: IR camera temperature reading of the process chamber of Haemoprocessor device post 1 min of actuation; Figure S5: Haemoprocessor trial summary for 10× and 40× dilution of Human Blood; Video SV1: Real-time video of 2.1 µm + 5 µm sorting at 192 MHz and 285 MHz; Video SV2: Real-time video of plasma separation from 20x diluted human blood at 285 MHz

## References

[1] Mielczarek W.S., Obaje E., Bachmann T., Kersaudy-Kerhoas M. (2016). Microfluidic blood plasma separation for medical diagnostics: Is it worth it?. Lab Chip.

[2] Heraud P., Chatchawal P., Wongwattanakul M., Tippayawat P., Doerig C., Jearanaikoon P., Perez-Guaita D., Wood B.R. (2019). Infrared spectroscopy coupled to cloud-based data management as a tool to diagnose malaria: A pilot study in a malaria-endemic country. Malar. J..

[3] Roy S., Perez-Guaita D., Bowden S., Heraud P., Wood B.R. (2019). Spectroscopy goes viral: Diagnosis of hepatitis B and C virus infection from human sera using ATR-FTIR spectroscopy. Clin. Spectrosc..

[4] Kochan K., Bedolla D.E., Perez-Guaita D., Adegoke J.A., Chakkumpulakkal Puthan Veettil T., Martin M., Roy S., Pebotuwa S., Heraud P., Wood B.R. (2021). Infrared Spectroscopy of Blood. Appl. Spectrosc..

[5] Dixon C., Lamanna J., Wheeler A.R. (2020). Direct loading of blood for plasma separation and diagnostic assays on a digital microfluidic device. Lab Chip.

[6] Sajeesh P., Sen A.K. (2014). Particle separation and sorting in microfluidic devices: A review. Microfluid. Nanofluid..

[7] Kar S., Maiti T.K., Chakraborty S. (2015). Capillarity-driven blood plasma separation on paper-based devices. Analyst.

[8] Hauser J., Lenk G., Hansson J., Beck O., Stemme G.R., Roxhed N. (2018). High-yield passive plasma filtration from human finger prick blood. Anal. Chem..

[9] Baillargeon K.R., Murray L.P., Deraney R.N., Mace C.R. (2020). High-Yielding Separation and Collection of Plasma from Whole Blood Using Passive Filtration. Anal. Chem..

[10] Yang X., Forouzan O., Brown T.P., Shevkoplyas S.S. (2012). Integrated separation of blood plasma from whole blood for microfluidic paper-based analytical devices. Lab Chip.

[11] Marques A., Veigas B., Araújo A., Pagará B., Baptista P.V., Águas H., Martins R., Fortunato E. (2019). Based SeRS platform for one-Step Screening of tetracycline in Milk. Sci. Rep..

[12] Berger A.G., Restaino S.M., White I.M. (2017). Vertical-flow paper SERS system for therapeutic drug monitoring of flucytosine in serum. Anal. Chim. Acta.

[13] Park M., Jung H., Jeong Y., Jeong K.-H. (2017). Plasmonic schirmer strip for human tear-based gouty arthritis diagnosis using surface-enhanced Raman scattering. ACS Nano.

[14] Hu S.-W., Qiao S., Pan J.-B., Kang B., Xu J.-J., Chen H.-Y. (2018). A paper-based SERS test strip for quantitative detection of Mucin-1 in whole blood. Talanta.

[15] Kersaudy-Kerhoas M., Dhariwal R., Desmulliez M.P., Jouvet L. (2010). Hydrodynamic blood plasma separation in microfluidic channels. Microfluid. Nanofluid..

[16] Yan S., Zhang J., Alici G., Du H., Zhu Y., Li W. (2014). Isolating plasma from blood using a dielectrophoresis-active hydrophoretic device. Lab Chip.

[17] Lenshof A., Ahmad-Tajudin A., Jarås K., Sward-Nilsson A.-M., Åberg L., Marko-Varga G., Malm J., Lilja H., Laurell T. (2009). Acoustic whole blood plasmapheresis chip for prostate specific antigen microarray diagnostics. Anal. Chem..

[18] Li P., Huang T.J. (2018). Applications of acoustofluidics in bioanalytical chemistry. Anal. Chem..

[19] Laurell T., Petersson F., Nilsson A. (2007). Chip integrated strategies for acoustic separation and manipulation of cells and particles. Chem. Soc. Rev..

[20] Faridi M., Ramachandraiah H., Iranmanesh I., Grishenkov D., Wiklund M., Russom A. (2017). MicroBubble activated acoustic cell sorting. Biomed. Microdevices.

[21] Meng L., Cui X., Dong C., Liu X., Zhou W., Zhang W., Wang X., Niu L., Li F., Cai F. (2020). Microbubble enhanced acoustic tweezers for size-independent cell sorting. Appl. Phys. Lett..

[22] Pei Z., Ma Y., Wang C., Wu Y., Song F., Wu X. (2021). Optimal design of a driver of interdigital transducers used to generate standing surface acoustic waves for cell sorting. Rev. Sci. Instrum..

[23] Ma Z., Zhou Y., Collins D.J., Ai Y. (2017). Fluorescence activated cell sorting via a focused traveling surface acoustic beam. Lab Chip.

[24] Mutafopulos K., Spink P., Lofstrom C., Lu P., Lu H., Sharpe J., Franke T., Weitz D. (2019). Traveling surface acoustic wave (TSAW) microfluidic fluorescence activated cell sorter (μFACS). Lab Chip.

[25] Ma Z., Collins D., Zhou Y., Ai Y. Fluorescence Activated Cell Sorting (FACS) System Based on Focused Traveling Surface Acoustic Waves (FTSAWs). Proceedings of the 7th International Multidisciplinary Conference on Optofluidics 2017.

[26] Lu H., Mutafopulos K., Heyman J.A., Spink P., Shen L., Wang C., Franke T., Weitz D.A. (2019). Rapid additive-free bacteria lysis using traveling surface acoustic waves in microfluidic channels. Lab Chip.

[27] Weser R., Winkler A., Weihnacht M., Menzel S., Schmidt H. (2020). The complexity of surface acoustic wave fields used for microfluidic applications. Ultrasonics.

[28] Liu G., Li Z., Li X., Li Y., Tang H., Wang M., Yang Z. (2020). Design and experiment of a focused acoustic sorting chip based on TSAW separation mechanism. Microsyst. Technol..

[29] Liu G., He F., Li Y., Zhao H., Li X., Tang H., Li Z., Yang Z., Zhang Y. (2019). Effects of two surface acoustic wave sorting chips on particles multi-level sorting. Biomed. Microdevices.

[30] Shiokawa S., Matsui Y., Ueda T. Liquid streaming and droplet formation caused by leaky Rayleigh waves. Proceedings of the IEEE 1989 Ultrasonics Symposium.

[31] Quintero R., Simonetti F. (2013). Rayleigh wave scattering from sessile droplets. Phys. Rev. E.

[32] Newton M., Banerjee M.K., Starke T., Rowan S.M., McHale G. (1999). Surface acoustic wave–liquid drop interactions. Sens. Actuators A Phys..

[33] Wang Y., Chen D., Wu C., Xie J. (2019). Effect of droplet boundary behaviors on SAW attenuation for potential microfluidic applications. Jpn. J. Appl. Phys..

[34] Alghane M., Fu Y.Q., Chen B., Li Y., Desmulliez M., Walton A. (2011). Streaming phenomena in microdroplets induced by Rayleigh surface acoustic wave. J. Appl. Phys..

[35] Collins D.J., Neild A., Ai Y. (2016). Highly focused high-frequency travelling surface acoustic waves (SAW) for rapid single-particle sorting. Lab Chip.

[36] Alghane M., Fu Y.Q., Chen B., Li Y., Desmulliez M.P.Y., Walton A. (2012). Frequency effect on streaming phenomenon induced by Rayleigh surface acoustic wave in microdroplets. J. Appl. Phys..

[37] Muller P.B., Barnkob R., Jensen M.J.H., Bruus H. (2012). A numerical study of microparticle acoustophoresis driven by acoustic radiation forces and streaming-induced drag forces. Lab Chip.

[38] Collins D.J., Ma Z., Ai Y. (2016). Highly localized acoustic streaming and size-selective submicrometer particle concentration using high frequency microscale focused acoustic fields. Anal. Chem..

[39] Jo M.C., Guldiken R. (2011). A label-free cell separation using surface acoustic waves. Annu. Int. Conf. IEEE Eng. Med. Biol. Soc..

[40] Nakashima Y., Hata S., Yasuda T. (2010). Blood plasma separation and extraction from a minute amount of blood using dielectrophoretic and capillary forces. Sens. Actuators B Chem..

[41] Nivedita N., Papautsky I. (2013). Continuous separation of blood cells in spiral microfluidic devices. Biomicrofluidics.

[42] Mathew B., Alazzam A., Destgeer G., Sung H.J. (2016). Dielectrophoresis based cell switching in continuous flow microfluidic devices. J. Electrost..

[43] Ung W., Mutafopulos K., Spink P., Rambach R., Franke T., Weitz D. (2017). Enhanced surface acoustic wave cell sorting by 3D microfluidic-chip design. Lab Chip.

[44] Garg N., Westerhof T.M., Liu V., Liu R., Nelson E.L., Lee A.P. (2018). Whole-blood sorting, enrichment and in situ immunolabeling of cellular subsets using acoustic microstreaming. Microsyst. Nanoeng..

[45] Sivanantha N. (2021). Novel Microfluidic Techniques to Evaluate Cell Adhesion Properties for Medical Applications. Ph.D. Thesis.

[46] Jolliffe I.T., Cadima J. (2016). Principal component analysis: A review and recent developments. Philos. Trans. R. Soc. A Math. Phys. Eng. Sci..

[47] Martin M., Perez-Guaita D., Andrew D.W., Richards J.S., Wood B.R., Heraud P. (2017). The effect of common anticoagulants in detection and quantification of malaria parasitemia in human red blood cells by ATR-FTIR spectroscopy. Analyst.

[48] Leon S., Shapiro B., Sklaroff D., Yaros M. (1977). Free DNA in the serum of cancer patients and the effect of therapy. Cancer Res..

[49] Serafin A., Malinowski M., Prażmowska-Wilanowska A. (2020). Blood volume and pain perception during finger prick capillary blood sampling: Are all safety lancets equal?. Postgrad. Med..

[50] Paraskevaidi M., Matthew B.J., Holly B.J., Hugh B.J., Thulya C.P., Loren C., StJohn C., Peter G., Callum G., Sergei K.G. (2021). Clinical applications of infrared and Raman spectroscopy in the fields of cancer and infectious diseases. Appl. Spectrosc. Rev..

[51] Veettil T.C.P., Kochan K., Edler K.J., De Bank P., Heraud P., Wood B.R. (2021). Disposable Coverslip for Rapid Throughput Screening of Malaria Using Attenuated Total Reflection spectroscopy. Appl. Spectrosc..

