# Supplementary material for: Haemoprocessor: A Portable Platform Using Rapid Acoustically Driven Plasma Separation Validated by Infrared Spectroscopy for Point-of-Care Diagnostics
Source: Biosensors (Basel). 2022 Feb 14;12(2):119. doi: 10.3390/bios12020119 (PMC8924765; doi:10.3390/bios12020119)
Supplement: Supplementary file 1 [file biosensors-12-00119-s001.zip › Haemoprocessor-Supplementary_Biosensors.pdf]

Supplementary Information

Haemoprocessor: A Portable Platform Using Rapid Acoustically Driven Plasma Separation Validated by Infrared Spectroscopy for Point-of-Care Diagnostics

KamalPrakash Prasanna Ravindran Nair [1,2,3], Thulya Chakkumpulakkal Puthan Veettil [4], Bayden R. Wood [4], Debjani Paul [2,3] and Tuncay Alan [1,3,*]

[1] Department of Mechanical & Aerospace Engineering, Monash University, Melbourne, VIC 3800, Australia; prasanna.nair@monash.edu

[2] Department of Biosciences & Bioengineering, Indian Institute of Technology Bombay, Mumbai 400076, India. debjani.paul@iitb.ac.in

[3] IIT Bombay—Monash Academy, Indian Institute of Technology Bombay, Mumbai 400076, India

[4] Monash Centre for Biospectroscopy, Department of Chemistry, Monash University, Melbourne, VIC 3800, Australia; thulya.chakkumpulakkalputhanveettil@monash.edu (T.C.P.V.); bayden.wood@monash.edu (B.R.W.)

* Correspondence: tuncay.alan@monash.edu (T.A.)

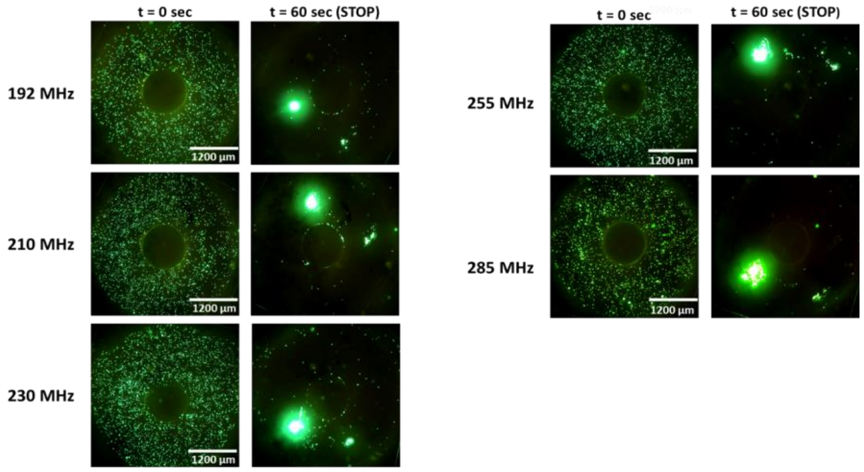

Figure S1. Frequency hopping (192 MHz – 285 MHz) summary 6 µm PS beads.

Figure S1: Depicting the process images of 6 µm PS beads at 192 MHz, 210 MHz, 230 MHz, 255 MHz, and 285 MHz. The trials were stopped at t = 60 seconds after a continuous run – note that the collection results were not very different whether it is run continuously or discretely.

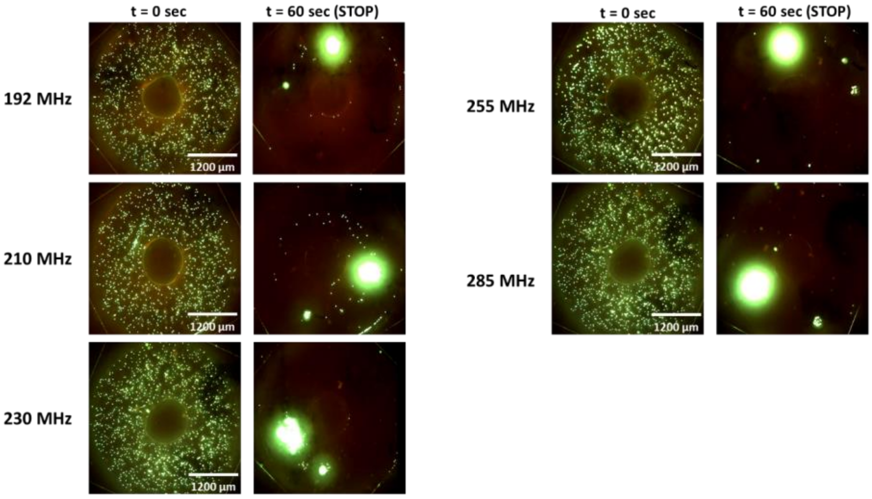

Figure S2. Frequency hopping (192 MHz – 285 MHz) summary 9.9 µm PS beads.

Figure S2: Depicting the process images of 9.9 µm PS beads at 192 MHz, 210 MHz, 230 MHz, 255 MHz, and 285 MHz. The trials were stopped at t = 60 seconds after a continuous run – note that the collection results were not very different whether it is run continuously or discretely.

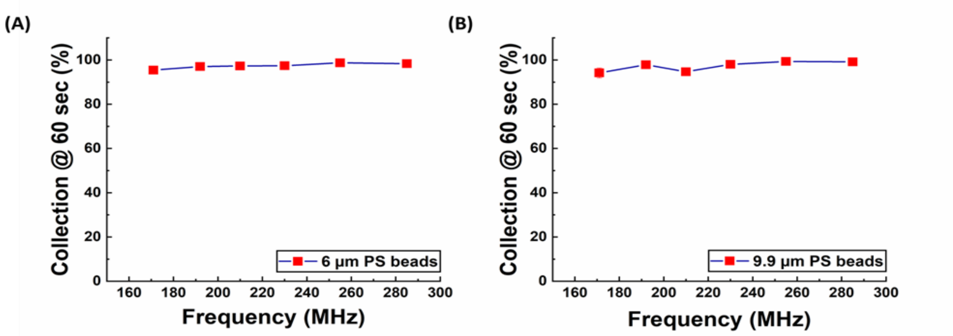

Figure S3. Frequency hopping (192 MHz – 285 MHz) collection (%) plots for 6 µm and 9.9 µm PS beads.

Figure S3: Depicting the collection (%) of the 6 µm and 9.9 µm particles at the end of 60 seconds for each frequency of actuation (192 MHz, 210 MHz, 230 MHz, 255 MHz, and 285 MHz). There was practically little difference in collection across the frequencies for both particles although proportionality of the collection (%) with the frequency of actuation was evident albeit marginal for particles of these sizes.

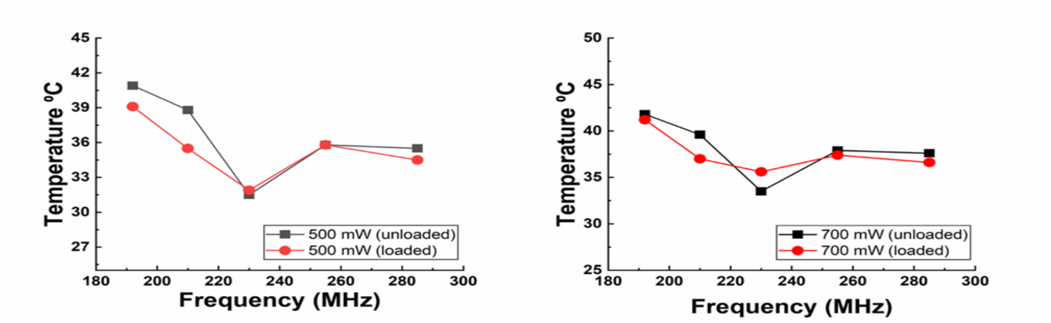

Figure S4. IR camera temperature reading of the process chamber of Haemoprocessor device post 1 min of actuation.

Figure S4: Depicting the temperature of the device after 60 seconds of actuation. The readings were made with DI water (loaded) and blank (unloaded) states. The loaded reading was always equal or less than the reading for unloaded - which was attributed to the evaporative cooling of the device in case of the former. While all the Polystyrene bead runs were done at 500 mW, the plasma separation was done at 0.63W although even at 700 mW, the temperature at 285 MHz is ca. 36.3°C which makes Haemoprocessor suitable for processing of human blood cells without the need for an external cooling apparatus.

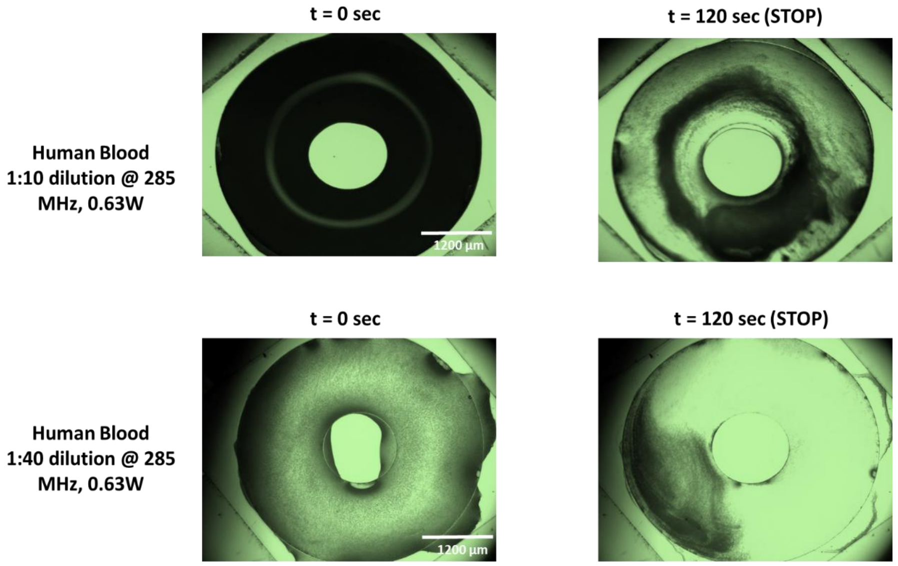

Figure S5. Haemoprocessor trial summary for 10X and 40X dilution of Human Blood.

Figure S5. Depicting the plasma separation process with Human blood at 1:10 (above) and 1:40 (below) dilution in PBS (pH 7.4). In case of the former, the device is overwhelmed by the number of cells as well as the viscosity while in the latter, the reduced viscosity results in the inability to the retain the mass of cells in the collection zone post-process – both of which (unlike the 1:20 dilution) compromises the ability of the device to deliver clear plasma. .
